# Medium-Intensity Treadmill Exercise Exerts Beneficial Effects on Bone Modeling Through Bone Marrow Mesenchymal Stromal Cells

**DOI:** 10.3389/fcell.2020.600639

**Published:** 2020-11-24

**Authors:** Lingli Zhang, Yu Yuan, Wei Wu, Zhongguang Sun, Le Lei, Jing Fan, Bo Gao, Jun Zou

**Affiliations:** ^1^School of Kinesiology, Shanghai University of Sport, Shanghai, China; ^2^Institute of Orthopedic Surgery, Xijing Hospital, Fourth Military Medical University, Xi’an, China

**Keywords:** treadmill exercise, bone morphogenetic protein, BMP-Smad signaling pathway, bone marrow mesenchymal stromal cell, bone formation, enzyme inhibitor

## Abstract

As a type of multipotential cells, bone marrow mesenchymal stromal cells (BMMSCs) can differentiate into chondrocytes, osteoblasts, and adipocytes under different loading condition or specific microenvironment. Previous studies have shown that BMMSCs and their lineage-differentiated progeny (for example, osteoblasts), and osteocytes are mechanosensitive in bone. The appropriate physical activity and exercise could help attenuate bone loss, effectively stimulate bone formation, increase bone mineral density (BMD), prevent the progression of osteoporosis, and reduce the risk of bone fractures. Bone morphogenetic protein (BMP) is originally discovered as a protein with heterotopic bone-inducing activity in the bone matrix that exerts a critical role in multiple stages of bone metabolism. In the present study, the medium-intensity treadmill exercise enhanced bone formation and increased *osteocalcin (OCN)* and *osteopontin (OPN)* mRNA expression as well as activation of the BMP-Smad signaling pathway *in vivo*. In order to investigate the effect of a BMP-Smad signaling pathway, we injected mice with activated enzyme inhibitors (LDN-193189HCL) and subjected the mice to treadmill exercise intervention. LDN-193189HCL attenuated the BMD and bone mass mediated by medium-intensity exercise and BMP-Smad signaling pathway.

## Introduction

Bone is critical for the sports system. The existence and structure of bone depends completely on the formation, quantity, and activity of bone cells that are regulated by several factors, such as mechanical and chemical stimuli, receptor and signal transduction, transcription and translation, heredity, nutrition, and endocrines (Rodan, 1998). Exercise or mechanical stimulation is one of the main factors affecting bone mass; it does not completely inhibit the loss of bone mineral content with increasing age and menopause and has a positive effect on several circulational index of bone metabolism (Smith et al., 2014). The appropriate physical activity and exercise could help attenuate bone loss, effectively stimulate bone formation, increase bone mineral density (BMD), prevent the progression of osteoporosis, and reduce the risk of bone fractures (Smith and Gilligan, 1991). The exercise can also reduce the risk of falls and increase the bone strength, flexibility, balance, and reaction time (Smith and Gilligan, 1991). Thus, exercise is known to improve bone health based on two aspects primarily by increasing the growth period of peak bone mass (PBM) in children and adolescents and maintaining or slowing down the aging (especially emphasizes the premenopausal women) with respect to bone absorption rate (Sumida et al., 2014; Winther et al., 2014; Tucker et al., 2015). Long-time high-intensity exercise or physical activity were bad for bone healthy, such as soldiers and marathon runners.

Recent studies have greatly expanded the knowledge of mechanoresponse of mesenchymal stem cells and their roles on the maintenance of bone homeostasis (Potier et al., 2010). Many studies in the mechanosensibility of bone marrow mesenchymal stromal cells (BMMSCs) have indicated central roles of BMMSCs in locomotion induced bone mass increase (Casazza et al., 2012; Shin et al., 2013; Monika et al., 2015). As a type of multipotential cells, BMMSCs can differentiate into chondrocytes, osteoblasts, and adipocytes under different loading condition (Zhang et al., 2016) or specific microenvironment. Previous studies have shown that BMMSCs and their lineage-differentiated progeny (for example, osteoblasts), and osteocytes are mechanosensitive in bone (Wang et al., 2020). And it is suggested that constant loading in an appropriate intensity is able to switch the differentiation direction of BMMSCs from adipogenesis to osteoblastogenesis (Casazza et al., 2012; Monika et al., 2015). Besides, the increase of BMMSC proliferation induced by fluid shear stress also contributes to the number of total bone cell population (Riddle et al., 2005). Accordingly, a direct connection between locomotion and enhancement of bone quality is built in light of the mechanical ability of BMMSCs. It is still unclear how these ubiquious signals arising from mechanical stimulation are perceived by BMMSCs and then how the cells respond to them. The key regulatory signals are generated during exercise and that these factors are first and foremost mechanical in nature.

The differentiation of BMMSCs toward an osteogenic lineage relies on various of signaling pathways including: Bone morphogenetic protein (BMP), IGF, NOTCH, Hedgehog and Wnt signaling (James, 2013). BMP is originally discovered as a protein with heterotopic bone-inducing activity in the bone matrix. BMPs are multi-functional growth factors that belong to the transforming growth factor beta (TGF-β) superfamily (Di et al., 2003). The signaling transduction by BMPs occurs specifically via both canonical Smad-dependent pathways (TGF-β/BMP ligands, receptors and Smads) and non-canonical Smad-independent signaling pathway (MAPK pathway) (Chen et al., 2012). Mechanical load can stimulate the activation of osteoblasts. Effective osteogenesis differentiation is highly dependent on the growth factor signals and mechanical load. It is still unclear how exercise type, intensity, duration, and frequency affect bone mass and strength. Hitherto, exercise and mechanical load promoted the expression of BMP-related target genes in osteoblasts and improved the morphological structure and mechanical properties of bone.

In order to investigate the effect of a BMP-Smad signaling pathway, we injected mice with activated enzyme inhibitors (LDN-193189HCL) and subjected the mice to treadmill exercise intervention. BMD, bone mass, the content of osteoblasts and osteoclasts, and the index of serum and urine were tested. In this study, we aimed to investigate the influence of exercise on bone metabolism in mice with or without the inhibition of BMP-Smad signaling pathway.

## Materials and Methods

### Treatment of Animals

**FIGURE 1:**
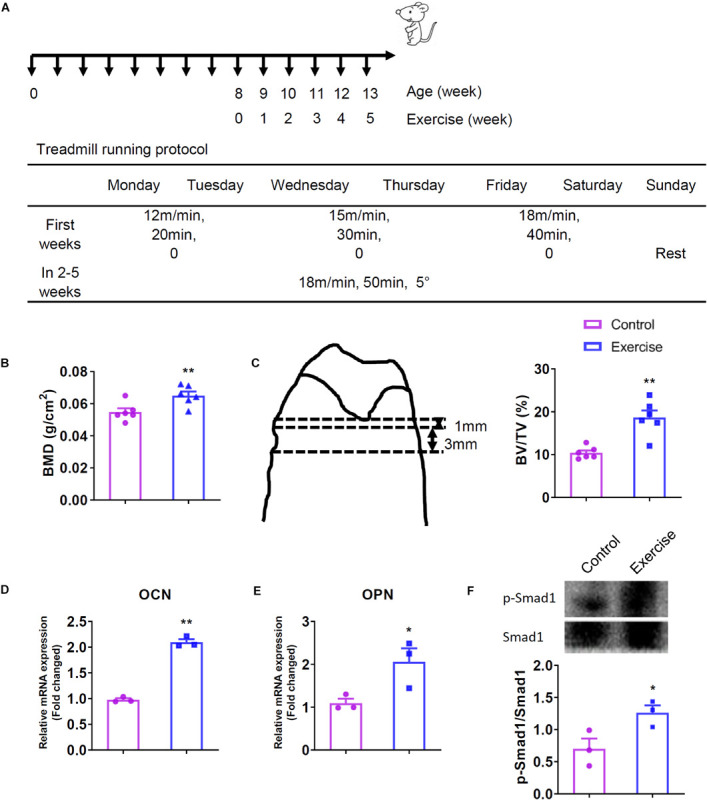
Exercise increased bone mineral density (BMD) and trabecular bone volume (BV/TV) of the femurs and activated bone morphogenetic protein signaling (BMPS) pathway. Mice were exercised for 5 weeks and then killed. The femurs of these mice were used for BMD and bone mass determination, and tibias were used for evaluating the mRNA expression and protein activation. **(A)** Treadmill running protocol. **(B)** BMD values of mice (*n* = 6). **(C)** The area of bone histomorphometry was used for calculating the bone mass and BV/TV values of mice (*n* = 6). **(D)** *OCN* mRNA expression of mice (*n* = 3). **(E)** *OPN* mRNA expression of mice (*n* = 3). **(F)** Activation of Smad1 proteins in mice (*n* = 3). *P < 0.05, **P < 0.01 as compared to the control group.

Twelve 2-month-old C57BL/6 male mice were randomly divided into two groups with six mice in each group: control group (Control), and exercise group (Exercise) in part one experiment. Exercise group was assigned treadmill running for 5 weeks, 6 days per week. The exercise program was adapted from previous studies (Mehl et al., 2005; Ferreira et al., 2007). Briefly, the mice were trained on a treadmill (Figure 1A). The speed of exercise group was equivalent to 85% maximal oxygen uptake (VO_2max_).

**FIGURE 2:**
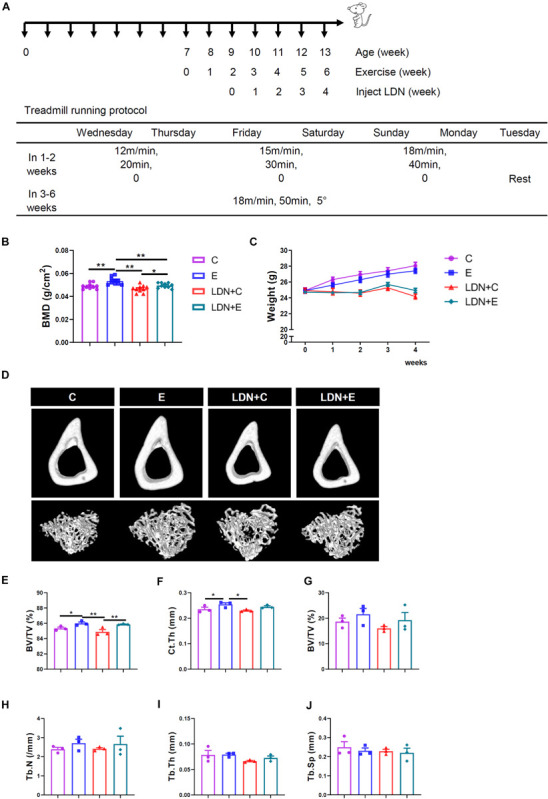
Effects of exercise and LDN injection on mouse body weight and bone mineral density (BMD) of the femurs. Exercise increased cortical bone volume (BV/TV), and cortical thickness (Ct.Th) of tibia. Mice were exercised for 6 weeks in the presence or absence of LDN and then killed. The femurs of these mice were used for BMD determination. Body weights of mice in the four groups were recorded every week during the exercise. **(A)** Treadmill running protocol. **(B)** BMD values of mice (*n* = 10). **(C)** Average body weight was shown (*n* = 18). **(D)** Representative μCT images showed two-dimensional cortical and trabecular architecture in mouse tibia (*n* = 3). **(E)** Cortical bone volume per tissue volume (BV/TV). **(F)** Cortical thickness (Ct.Th). **(G)** Trabecular bone volume per tissue volume (BV/TV). **(H)** Trabecular number (Tb.N). **(I)** Trabecular thickness (Tb.Th). **(J)** Trabecular separation (Tb.Sp). *P < 0.05, **P < 0.01.

Seventy-two 7-week-old C57BL/6 male mice were divided into four groups in accordance with weight and BMD of mice with 18 mice in each group: control group (C), exercise group (E), LDN group (LDN+C), and exercise with LDN group (LDN+E) in part two experiment. Mice in the exercise group and exercise with LDN group were assigned treadmill running exercise at intensities for 6 weeks, 6 days per week (Figure 2A).

All mice were maintained at five mice per cage, and provided food and water *ad libitum*. All mice were housed in an environment maintained on a 12-h light–dark cycle at 22 ± 2°C. All the mice were anesthetized with ether inhalation the animals were euthanized by cervical vertebra dislocation.

### LDN-193189HCL Injection

Mice in the LDN groups were injected with LDN (LDN-193189HCL, Selleckchem) on Wednesday from the third to sixth week by intraperitoneal injection (Yu et al., 2008a; Lee et al., 2011; Katagiri and Tsukamoto, 2013). LDN (3 mg/kg body weight) was mixed with DMSO and slowly added this DMSO liquor into physiological saline. LDN powder was completely dissolved using water bath ultrasonic and dissolved by 0.22 μm filter. Each mouse was weighed before injecting and the doses of LDN were adjusted.

### Calcein Injection

A 20.0 mg calcein was mixed with 5 ml 4% NaHCO_3_ (PH: 8.0–8.5) and 5 ml stroke-physiological saline solution. Mouse bones were labeled with subcutaneously injected calcein (5 μl/g) at day 1 and 8 before the mice were killed at day 9.

### BMMSCs Cultures and ALP Staining

The femurs of mice were used for BMMSCs extraction and culture. The bone marrow cells were washed out with α-MEM (HyClone, United States). After being centrifuged at 1000 rpm for 10 min, the pellets were resuspended in α-MEM supplemented with 10% fetal bovine serum (FBS) (Gibco, United States), and 1% penicillin-streptomycin (Invitrogen, United States). The cells were seeded at a density of 5 × 10^6^ cells/well in 12-well plates. Alkaline phosphatase (ALP) staining was performed 7 days after seeding (Sigma-Aldrich ALP stain kit, United States), and images were captured using a microscope (Nikon Eclipse 90i and Nikon Elements AR 3 software, Japan). Colony-forming units (CFUs) were calculated from those images.

### BMD Measurement of Total Femurs

The left femurs were used for BMD. Special attention was paid to avoiding damage of the bone cortex when removing the skin, muscles, and connective tissues. Before testing, the specimens were observed under a microscope to make sure all specimens were intact. The dual energy X-ray absorptiometry method was used for measurement of BMD (Osteocore3 Digital 2D, France) of the total left femurs.

### Bone Histomorphometry

The right femurs were isolated, fixed in 4% paraformaldehyde (PFA) after washing with PBS, and then dehydrated in 70% ethanol. The specimens were dehydrated sequentially in ascending concentrations of ethanol (95%, 100%) and xylene, and then embedded in methyl methacrylate. The femurs were cut at 4-μm thickness using a microtome (Leica RM2265, Germany), transferred onto gelatin-coated slides, and dried overnight in an oven at 60°C. After the slices were stained using Masson’s method, the digitizing morphometric system (Osteomeasure High Resolution Color Subsystem) was used to measure the bone histomorphometric parameters. In the present study, the region of cancellous bone measured was 1–4 mm distal to the lower margin of the growth plate in the proximal femur (Figure 1C). The measured parameters for cancellous bone included bone volume (BV), the total tissue volume (TV), bone surface (BS), single-labeled surfaces (sLS), double-labeled surfaces (dLS) and so on. These data were used to calculate the percent cancellous mineral apposition rate (MAR), bone formation rate/bone volume (BFR/BV), bone formation rate/total tissue volume (BFR/TV), bone formation rate/bone surface (BFR/BS), bone volume (BV/TV), trabecular number (Tb.N), trabecular thickness (Tb.Th), and trabecular separation (Tb.Sp).

### μCT for BMD and Bone Histomorphometry

The right tibias were observed under a microscope to ensure all specimens were intact. After fixation with 4% PFA and dehydrated in 70% ethanol, the specimens were scanned by a μCT scanner (Skyscan 1174; Bruker, Kontich, Belgium) with acquisition settings of: 40 kVp, 555 uA, filter AL 0.5 mm, and 9 micron isotropic resolution. Bone microarchitecture parameters of cortical bone and trabecular bone were measured using the built-in software as follows: bone volume (BV/TV), cortical thickness (Ct.Th), Tb.N, Tb.Th, and Tb.Sp.

### Methods for Bone Biomechanics Measurement

The three-point bending method was performed for biomechanics analysis (Reger RGM-2020, China) immediately after BMD measurement. Then, a vernier caliper was used for measurement of femoral short axis, and minor axis in the central part of the direction of Tb.Th. When testing, the proximal and distal ends of the femur were fixed on the machine pedestal, with a 12-mm span and specimens in horizontal position. The probe moved continuously down at a speed of 1 mm/min for bending test with no preload. Data including maximum load, yield stress, and elastic modulus were automatically calculated by a computer program.

### Real-Time PCR

The total RNA was isolated from the left tibias of mice using TRIzol (Sigma, United States) and reverse-transcription (Revert Aid first strand cDNA Synthesis Kit, Cat.#:K1622, Thermo Scientific, United States) was conducted using 1 μg of total RNA with oligo-dT primers at 42°C for 1 h. All PCRs were performed using 2 μg of respective cDNA with SYBR Green qPCR Master Mix (Roche, Switzerland). *Osteocalcin (OCN)* mRNA, *osteopontin (OPN)* mRNA, *Osterix (OSX)* mRNA, *runt related transcription factor 2 (Runx2)* mRNA, *msh homeobox-2 (MSX2)* mRNA, *distal-less homeobox 5 (DLX5)* mRNA, and *GAPDH* mRNA were measured by quantitative testing. Quantification of mRNA was performed using Step One Plus^TM^ Sequencing Detection System (Life Technologies, United States). The mouse primers used in this study are presented in Table 1.

**TABLE 1:** RT-PCR primer Sequences.

**mRNA**	**Primer**	**Sequences (5′–3′)**
OCN	Forward	AGCAGGAGGGCAATAAGGTAGT
	Reverse	ACCGTAGATGCGTTTGTAGGC
OPN	Forward	CAGCCTGCACCCAGATCCTA
	Reverse	GCGCAAGGAGATTCTGCTTCT
OSX	Forward	TGGTACAAGGCAGGCATCCA
	Reverse	GGAGCAAAGTCAGATGGGTAAGT
Runx2	Forward	TTTAGGGCGCATTCCTCATC
	Reverse	TGTCCTTGTGGATTAAAAGGACTTG
MSX2	Forward	AACACAAGACCAACCGGAAG
	Reverse	GCCGTATATGGATGCTGCTT
DLX5	Forward	CACCACCCGTCTCAGGAATC
	Reverse	GCTTTGCCATAAGAAGCAGAGG
GAPDH	Forward	CCACAGTCCATGCCATCAC
	Reverse	CATACCAGGAAATGAGCTTGAC

### Western Blot Assay

The total proteins were extracted from other tibias with RIPA Lysis Buffer (Biyuntian, China) containing a protease inhibitor cocktail (Roche, Switzerland). Proteins were separated by 10% SDS-PAGE with 80 to 120 V, and then transferred onto a 0.45 μm PVDF membrane (Millipore, United States) with 300 mA for 2 h. The blotting membranes were blocked with 5% skim milk powder in 1 × TBS-Tween (TBST) for 1 h. After washing with 1 × TBST three times for 10 min once, the blotting membranes incubated with phospho-Smad1 (CST, #9553), Smad1 (CST, #9743), phospho-p44/42 MAPK (CST, #5726), p44/42 MAPK (CST, #9102), phospho-p38 MAPK (CST, #9211), p38 MAPK (CST, #9212), and β-Actin (CST, #5057). The molecular weight and gray value of the target strip were analyzed by the Image J system.

### Serum and Urine Biochemical Maker Analysis

Massage technique was used to obtain urine samples, which were stored at −80°C until further analysis. All the mice were anesthetized with ether inhalation; after blood samples were collected, the animals were euthanized by cervical vertebra dislocation. The serum samples were collected from the blood. The levels of serum calcium and phosphorus were measured by colorimetric assays (Total Calcium LiquiColor kit: Stanbio, TX, United States; inorganic phosphorus mensuration reagent kit: Shanghai, China). Urinary deoxypyridinoline (DPD) levels were measured by enzyme-linked immunosorbent assay (DPD EIA kit; METRA, San Diego, CA, United States). Urinary carboxy-terminal collagen cross-linking telopeptide of type I collagen (CTX-I) levels were measured by enzyme-linked immunosorbent assay (RatLaps^TM^ CTX-I EIA kit; Boldon, United Kingdom). Urinary creatinine (UCr) levels were measured by creatinine colorimetric assay using a creatinine mensuration reagent kit (Hubei, China).

### Serum Starvation

The mouse embryo fibroblasts (MEFs) were extracted from embryo of C57BL/6 mice (Anna et al., 2014), 14–16 days pregnant. Embryo washed three times with PBS. After being detached with 0.25% trypsin-EDTA (Gibco, United States) for 30 min, the pellets were resuspended in high-MEM supplemented with 10% FBS (Gibco, United States), and 1% penicillin-streptomycin (Invitrogen, United States) at 37°C in 5%CO_2_ incubator. The cells were seeded in six-well plates. When cells reached 80–90% confluence, the essential medium was changed to high-MEM supplemented 1% penicillin-streptomycin without FBS for 4 h. After 4 h, four six-well plates were divided into four groups with six wells in each group, then supplemented with 1% penicillin-streptomycin and mice serum in different groups, respectively. After 30 min, the cells were collected and used for measuring the phospho-Smad1, Smad1, and β-Actin. After 1 h, another cells were collected and used for measuring the phospho-p44/42 MAPK, p44/42 MAPK, phospho-p38 MAPK, p38 MAPK, and β-Actin.

### Statistical Analysis

All the data were expressed as mean ± SEM. Two-way analysis of variance (ANOVA) was performed, and statistical differences were calculated using independent sample *T*-test. Values of *P* < 0.05 were considered statistically significant. All statistical analysis was performed by statistical software SPSS 13.0 for Windows (version 13, SPSS, Chicago, IL, United States).

## Results

### Treadmill Exercise Increases BMD, Bone Volume of 2-Month-Old Male Mice and Activates BMP-Smad Signaling Pathway

In the present study, 2-month-old male mice undergo 5-week treadmill exercise were analyzed for BMD and bone histomorphometry. We found that treadmill exercise significantly improved bone mass and quality including BMD and BV/TV compared to control littermates (Figures 1B,C), and was efficient to up-regulated the expression of *OCN* and *OPN* (Figures 1D,E). Mechanistically, western blot analysis showed that exercise activated the Smad1 protein phosphorylation (Figure 1F).

### Treadmill Exercise Increases BMD, Cortical Bone BV/TV and Ct.Th of Tibia, and LDN Injection Shows a Significant Effect on Mouse Body Weight

To further investigate the role of BMP signaling in treadmill exercise-induced bone formation, 7-week-old male mice were performed a 6-week round exercise and injected specific BMP singnaling inhibitor – LDN. The mice ran at the speed of 18 m/min on a 5° slope, which is categorized as medium-intensity exercise. We found that 6 weeks of medium-intensity exercise significantly increased mice BMD. BMD in the E group was significantly higher than that in the C group (*P* < 0.01), and BMD in the LDN+E group was higher than that in the LDN+C group (*P* < 0.05); four times inhibition of BMP signaling decreased the BMD that was significantly lower in the LDN+E group than that in the E group (*P* < 0.01) (Figure 2B). Mouse body weight was measured every week during exercise and inhibition of BMP signaling (Table 2 and Figure 2C). Inhibition of BMP signaling decreased the mouse body weight; however, the exercise did not show a significant effect on mouse body weight during Inhibition of BMP signaling for 3 weeks. These results suggested that medium-intensity exercise increased the BMD in mice, whereas LDN attenuated the BMD.

**TABLE 2:** Effects of exercise and LDN on mouse body weight.

**Group**	***N***	**Weight (g)**				
		**0**	**1**	**2**	**3**	**4**
C	18	24.92 ± 1.43	26.31 ± 1.33	26.94 ± 1.62	27.42 ± 1.68	28.07 ± 1.73
E	18	24.92 ± 1.13	25.61 ± 1.09	26.28 ± 1.46	27.00 ± 1.55	27.41 ± 1.24
LDN+C	18	24.91 ± 1.18	24.79 ± 1.68**	24.58 ± 1.63**	25.27 ± 1.45**	24.17 ± 1.39**^△△^
LDN+E	18	24.72 ± 0.33	24.64 ± 0.57^△△^	24.67 ± 0.95^△△^	25.70 ± 0.97^△^	24.93 ± 1.13**^△△^

**P < 0.01 as compared to the C group. ^△^*P* < 0.05, ^△△^*P* < 0.01 as compared to the E group.

Next, exercise was found to increase the cortical bone BV/TV and Ct.Th of tibia (Figure 2D): cortical bone BV/TV in the E group was higher than that in the C group (*P* < 0.05), cortical bone BV/TV in the LDN+E group was significantly higher than that in the LDN+C group (*P* < 0.01) (Figure 2E); whereas, the Ct.Th in the E group was higher than that in the C group (*P* < 0.05) (Figure 2F). Inhibition of BMP signaling alone did not have any effect on the bone mass of tibia (Figures 2G–J). The data of three-point bending experiment including maximum load, yield stress, and elastic modulus were no difference between groups (Supplementary Figures S1A–C).

### Medium-Intensity Exercise Increases Bone Formation and Inhibits Bone Resorption, and LDN Injection Inhibits the Exercise–Mediated Bone Formation and Promotes Bone Resorption

**FIGURE 3:**
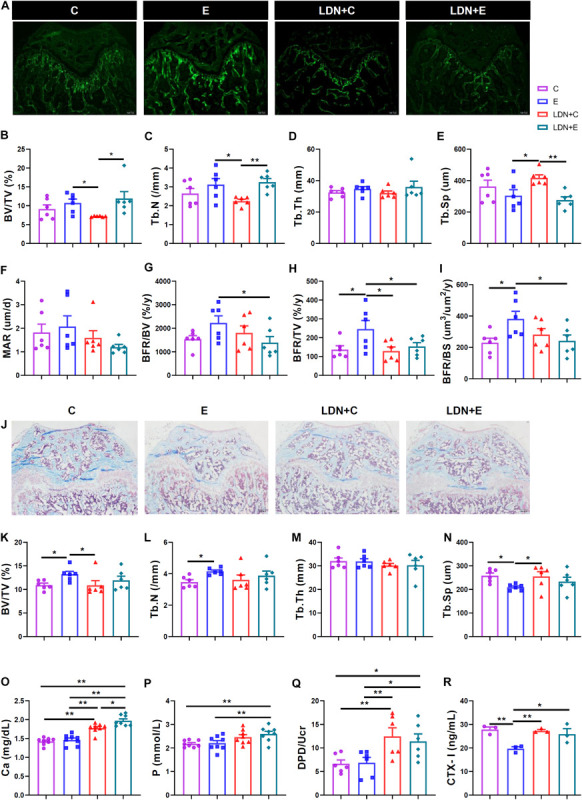
Medium-intensity exercise increased bone formation and inhibited bone resorption, and LDN injection inhibited the exercise–mediated bone formation and promoted bone resorption. Mice were exercised for 6 weeks in the presence or absence of LDN and then killed. **(A)** Mouse bones were labeled with subcutaneously injected calcein (5 μl/g) at days 1 and 8 (*n* = 6). **(B)** Trabecular bone volume (BV/TV). **(C)** Trabecular number (Tb.N). **(D)** Trabecular thickness (Tb.Th). **(E)** Trabecular separation (Tb.Sp). **(F)** Mineral apposition rate (MAR) values of the four groups of mice. **(G)** Bone formation rate/bone volume (BFR/BV) values of the four groups of mice. **(H)** Bone formation rate/total tissue volume (BFR/TV) values of the four groups of mice. **(I)** Bone formation rate/bone surface (BFR/BS) values of the four groups of mice. **(J)** After the slices were stained using Masson’s method, the femur bones of six mice were used for bone histomorphometry analysis (*n* = 6). The photos of the masson staining. **(K)** Trabecular bone volume (BV/TV). **(L)** Trabecular number (Tb.N). **(M)** Trabecular thickness (Tb.Th). **(N)** Trabecular separation (Tb.Sp). **(O)** The serum calcium level (*n* = 8). **(P)** The serum phosphorus level (*n* = 8). **(Q)** The urinary deoxypyridinoline (DPD) level (*n* = 6). **(R)** The urinary carboxy-terminal collagen cross-linking telopeptide of type I collagen (CTX-I) level (*n* = 3). *P < 0.05, **P < 0.01.

Mouse bones were labeled with subcutaneously injected calcein (5 μL/g) at days 1 and 8. After the slices were made, the bone volume was examined using bone histomorphometry (Figure 3A). We found that exercise increased the bone formation and LDN injection inhibited the exercise-based improvement in the bone formation. In addition, exercise increased the bone formation of femur: BV/TV and Tb.N in the LDN+E group were higher than those in the LDN+C group (Figures 3B,C), Tb.Sp in the LDN+E group was lower than that in the LDN+C group (Figure 3E), BFR/TV and BFR/BS in the E group were higher than those in the C group (Figures 3H,I). Also, we found that LDN injection inhibited the exercise-improved bone formation: BFR/BV, BFR/TV, and BFR/BS were lower in the LDN+E group than the E group (*P* < 0.05) (Figures 3G,I). Mice that were exercised for 6 weeks or injected with LDN alone revealed that BV/TV, Tb.N, and BFR/TV in the LDN+C group were lower than those in the E group (Figures 3B,C,H), while Tb.Sp in the LDN+C group was higher than that in the E group (Figure 3E). Tb.Th and MAR were no statistical differences (Figures 3D,F).

After the slices were stained using Masson’s method, the femur bones of 6 mice were used for bone histomorphometry analysis (Figure 3J). Consequently, exercise could increase the bone formation of femur: BV/TV and Tb.N in the E group were higher than those in the C group (Figures 3K,L), while Tb.Sp in the E group was lower than that in the C group (Figure 3N). Moreover, BV/TV in the LDN+C group was lower than that in the E group (Figure 3K), while Tb.Sp in the LDN+C group was higher than that in the E group (Figure 3N) when mice were exercised for 6 weeks or injected with LDN alone. Tb.Th was no statistical difference (Figure 3M).

We measured the calcium and phosphorus levels in the serum and found that inhibition of BMP signaling increased these levels. The serum calcium level in the LDN+C group was significantly higher than that in the C group (*P* < 0.01) (Figure 3O), while the serum calcium and phosphorus levels were significantly higher in the LDN+E group than that in the E group (*P* < 0.01) (Figures 3O,P). The serum calcium level in the LDN+E group was significantly higher than that in the LDN+C group (*P* < 0.05) (Figure 3O). Importantly, we found that medium-intensity exercise significantly decreased circulating DPD and CTX-I level, indicating the inhibition effect of bone resorption potency. Moreover, Inhibition of BMP signaling could attenuate the inhibitory effect of medium-intensity exercise, indicating BMP signaling as the key modulator of treadmill exercise-induced bone remoldling. Also, the urinary DPD and CTX-I levels, two *in vivo* bone resorption markers, were measured. Exercise led to a decrease in the urine CTX-I levels, while Inhibition of BMP signaling promoted the bone resorption. DPD/Ucr in the LDN+C group was significantly higher than that in the C group (*P* < 0.01) (Figure 3Q); DPD/Ucr and CTX-I levels were higher in the LDN+E group than the E group (*P* < 0.05) (Figures 3Q,R).

### Treadmill Exercise Activates Smad1 Protein Phosphorylation, and LDN Injection Inhibits the Expression of Osteoblast–Related Genes in Tibia

**FIGURE 4:**
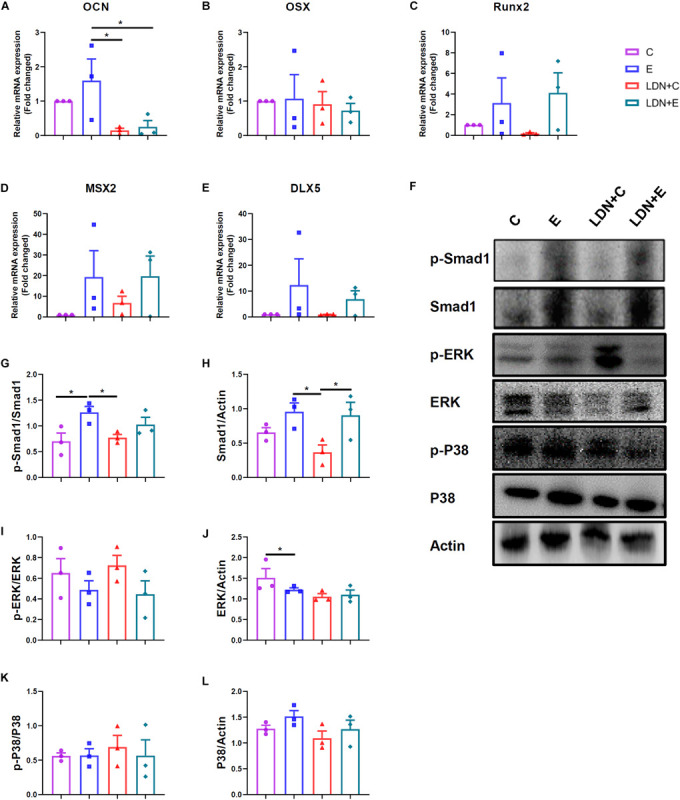
Exercise could active Smad1 protein, and LDN injection inhibited the expression of osteoblast–related genes in tibia. Mice were exercised for 6 weeks in the presence or absence of LDN and then killed. The left tibias were used for the evaluation of gene expression and protein activation. **(A)** The mRNA expression of *OCN* (*n* = 4). **(B)** The mRNA expression of *OSX* (*n* = 4). **(C)** The mRNA expression of *Runx2* (*n* = 4). **(D)** The mRNA expression of *MSX2* (*n* = 4). **(E)** The mRNA expression of *DLX5* (*n* = 4). **(F)** Representative images of protein expression by Western blot. **(G,H)** Western blot analysis with antibodies to p-Smad1 and Smad1 (*n* = 4). **(I,J)** Western blot analysis with antibodies to p-ERK and ERK (*n* = 4). **(K,L)** Western blot analysis with antibodies to p-P38 and P38 (*n* = 4). *P < 0.05.

The mRNA expression of *OCN* was lower in the LDN+E group than the E group (*P* < 0.05) (Figure 4A). The mRNA expression of OSX, Runx2, MSX2, DLX5 were no statistical differences (Figures 4B–E). The left tibias were used for the evaluation of protein activation (Figure 4F). Western blot analysis with antibodies to p-Smad1 and Smad1 showed that p-Smad1 expression was higher in the E group than in the C group (*P* < 0.05) (Figure 4G), indicating that exercise could activate the Smad1 protein phosphorylation. Also, Smad1 expression was higher in the LDN+E group than the LDN+C group (*P* < 0.05) (Figure 4H), suggesting that exercise improves Smad1 protein expression. ERK expression was lower in the E group than the C group (*P* < 0.05) (Figure 4J). The ERK and P38 protein phosphorylation, P38 expression were no statistical differences (Figures 4I,K,L).

### Medium Level of Exercise Increases Osteogenic Differentiation of BMMSCs and Activates Smad1 Protein Phosphorylation, and LDN Injection Inhibits Smad1 Protein Phosphorylation

**FIGURE 5:**
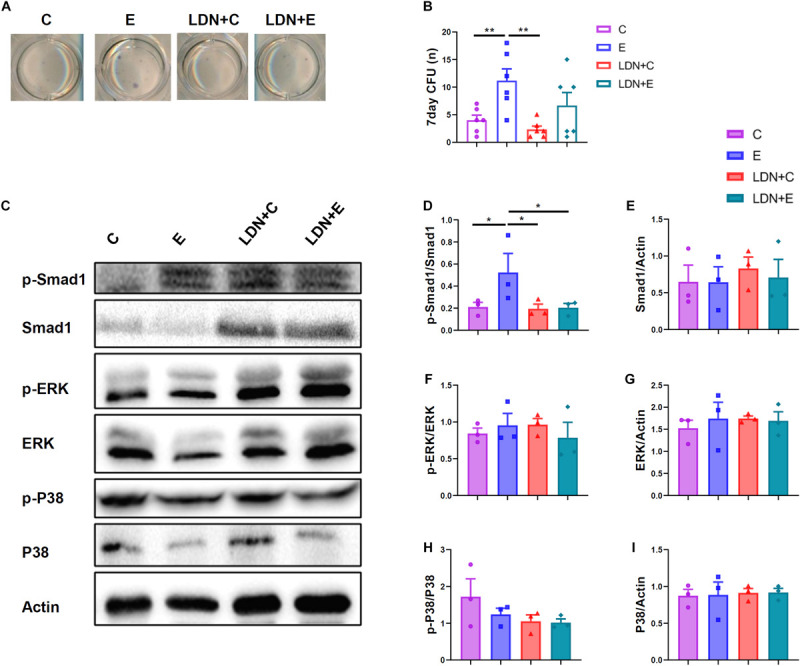
Effects of exercise and LDN injection on the number of BMMSCs, and exercise activated Smad1 protein phosphorylation, and LDN injection inhibited Smad1 protein phosphorylation. Mice were exercised for 6 weeks in the presence or absence of LDN and then killed. The bone marrow cells were flushed out, plated, and cultured for 10 days, and then stained for ALP. **(A)** Representative images of colony-forming units (CFU) staining. **(B)** ALP quantitation results (*n* = 6). **(C)** The mouse embryo fibroblasts (MEF) were extracted from embryo of C57BL/6 mice, 14–16 days pregnant. The essential medium was changed to high-MEM without FBS, supplemented with mice serum in different groups, respectively. After 30 min, the cells were used for measuring the protein activity. Representative images of protein expression by Western blot. **(D,E)** Western blot analysis with antibodies to p-Smad1 and Smad1. **(F,G)** Western blot analysis with antibodies to p-ERK and ERK. **(H,I)** Western blot analysis with antibodies to p-P38 and P38. *P < 0.05, **P < 0.01.

To further test whether medium level treadmill exercise have impact on BMMSCs, we isolated BMMSCs followed by previously described protocal and analyzed their colony-forming and osteogenic ability. ALP staining showed that medium level treadmill exercise markedly increase osteogenic differentiation potential. What’s more, inhibition of BMP signaling significantly attenuated. The ALP-positive CFU was measured, which indicated the number of osteogenic BMMSCs in the bone marrow. The medium level of exercise increased the number of osteogenic BMMSCs (Figures 5A,B).

Mouse embryo fibroblasts were extracted from embryo of C57BL/6 mice, 14–16 days pregnant. The essential medium was changed to high-MEM without FBS for 4 h, supplemented with mice serum in different groups, respectively. After 30 min, the cells were used for measuring the protein activity (Figure 5C). Exercise activated Smad1 protein phosphorylation, whereas LDN injection inhibited the Smad1 protein phosphorylation (Figures 5D,E). In addition, Inhibition of BMP signaling did not affect the phosphorylation of P38 and ERK proteins (Figures 5F–I).

## Discussion

The medium level exercise increased BMD and BV/TV of the femurs of 2-month-old male mice, in addition, increased the expression of osteoblast-related genes (*OCN* and *OPN*), and activated the BMP-Smad signaling pathway *in vivo*. OCN collagen constitutes osteoblast synthesis and secretion and is a specific marker of bone formation (Magni et al., 2016). OPN can stimulate the osteoblast proliferation and calcification, promote the adhesion of osteoclasts and bone matrix, and improve the bone resorption (Zhang et al., 2018). BMP signaling is one of the essential pathways involved in osteogenic differentiation of BMMSCs and regulation of bone formation (Huang et al., 2018). Therefore, we hypothesized that BMP-Smad signaling pathway mediates the treadmill exercise that can regulate BMMSCs to osteoblast differentiation in mice.

Mice were exercised for 6 weeks. Firstly, at the end of 2-week exercise training, the mice body weight of the four groups did not differ significantly. This exercise training was categorized as medium-intensity exercise, and the strength was about 85% VO_2max_ (Droste et al., 2003; Kramer et al., 2006; Petzinger et al., 2013). The results suggested that the mice can adapt to medium-intensity treadmill exercise for 4 weeks, which is similar to that reported previously (Zhang et al., 2017). In addition, the medium level of exercise could activate Smad1 protein phosphorylation from serum starvation experiment *in vitro*; the experimental results were in agreement with previous studies. However, it did not activate P38 and ERK protein phosphorylation.

Previous studies have shown that resistance exercise stimulates bone formation and only slightly affects bone resorption (Smith et al., 2008, 2014) as exercise renders muscle tension via the mechanical stress pathways and stimulates the bone formation. But running exercise could increase the BMD of growing mice (Huang et al., 2003). After 6 weeks of exercise, the medium level of exercise significantly increased the BMD, the cortical bone volume, and Ct.Th of tibia. Furthermore, bone histomorphometry showed that the medium level of exercise led to an increase in BFR/TV, BFR/BS, trabecular bone volume, and Tb.N, accompanied by a decrease in bone separation. These results are consistent with those from previous studies, which showed that medium-intensity exercise enhanced the bone formation (Ronga et al., 2013; Smith et al., 2014; Iura et al., 2015; Li et al., 2015). There was no significant difference in maximum load, yield stress, and elastic modulus of femurs between groups, which may be due to the stable biomechanical properties of bone. Bone strength mainly includes bone mass and bone quality. Although bone mass determines about 80% of bone biomechanical strength, recent data show that BMD can’t completely provide bone biomechanical index (Hasegawa et al., 2001). In addition to bone mass, it is related to the internal structure of bone such as the three-dimensional architecture of bone and the connectivity between trabeculae. Due to the stability of bone biomechanical properties, the effect of external intervention is not significant.

In addition, the ALP-positive CFU, an indication of the number of osteogenic BMMSCs in the bone marrow. Mouse ALP is frequently measured and used as a bone marker in mammalian bone research (Halling et al., 2013). The osteoblasts activity is improved with increased ALP secretion (Shen et al., 2007). Exercise stimulates the ALP production by osteogenic cells in order to increase the mineralization of the newly forming bone (Roussignol et al., 2012; Wang et al., 2018). Supposedly, the increase in bone formation and the number of osteoblasts may be mediated by the increase in the number of osteogenic BMMSCs (Zhang et al., 2017). Osteoclasts constantly resorb old bone, and osteoblasts form osteoid that is converted into mineralized bone. The urinary levels of DPD and CTX-I were bone resorption markers *in vivo* (Woitge et al., 1998; Vesper et al., 2002; Carvalho et al., 2006), and exercise leads to a decrease in the urinary levels of CTX-I. Previous studies have shown that exercise decreases the bone resorption (O’Kane et al., 2006; Kishimoto et al., 2012; Pacheco-Costa et al., 2018). These results indicated that medium level exercise suppressed bone resorption, which contributed to the increase in bone mass.

Bone morphogenetic proteins exert their diverse biological effects through two types of transmembrane receptors: BMP receptor type I (BMPR-I) and type II (BMPR-II) possess intrinsic serine/threonine kinase activity (Heldin et al., 1997). Type I and II BMP receptors are highly homologous transmembrane serine/threonine kinases. Only when two type I and two type II receptors form the heterogeneous dimers in the BMP family, they exhibit a high affinity toward the ligand. An allele-specific RNAi has been established for the mutant type I receptor, ALK2 (Lowery and Rosen, 2012). LDN-193189HCL as a BMP signaling inhibitor have been developed for the specific inhibition of BMP type I receptor-Smad1/5/8 signaling axis (Katagiri and Tsukamoto, 2013; Yu et al., 2017), inhibited the kinase ALK2 (the amino acid sequence of ALK2 login NP001103675) in mice (Lowery and Rosen, 2012). Consecutively, LDN-193189 is a potent derivative of dorsomorphin, the first chemical inhibitor explicitly identified for the receptor-induced phosphorylation of Smad1/5/8s but not the MAP kinase pathway (Yu et al., 2008b; Katagiri and Tsukamoto, 2013).

Inhibition of BMP-Smad signaling reduced the body weight in mice. Thus, LDN injection might potentially lead to weight loss by reducing bone mass. Because LDN-193189 has been shown to reduce the heterotopic bone formation in a transgenic mouse model carrying a mutant *ALK2* gene (Yu et al., 2008a; Katagiri and Tsukamoto, 2013). Furthermore, the pharmacological dose of LDN led to a slight decrease in BMD. The LDN injection further decreased the BFR/BV, BFR/TV, and BFR/BS in combination with the medium level of exercise in young mice. Moreover, the injection also decreased the osteoblast-related *OCN* mRNA expression. Activated Smads regulate the expression of transcriptional factors and transcriptional co-activators vital for osteoblasts differentiations (DLX5, Runx2, and OSX) (Chen et al., 2012). Herein, we have identified the altered expression of the BMP/Smad target gene, *DLX5*, which is an osteoblast-specific and BMP signaling specific transcription factor (Lee et al., 2003), critical for skeletal tissue development (Komori, 2019) and osteoblast differentiation (Tadic et al., 2002). Msx2 is the BMP/Smad target gene (Jovanovic et al., 2018), a homeodomain transcription factor relevant to osteoblast survival, programming (Tong and Lee, 2013), and orthotopic craniofacial bone formation (Jabs et al., 1993). The trend of *MSX2* gene expression was similar to that of *DLX5* and did not differ significantly. In addition, inhibition of BMP signaling increased the serum calcium and phosphorus levels, the bone absorption may be enhanced. The serum calcium and phosphorus levels were critical bone mineral components of the blood (Lin et al., 2012; Orcy et al., 2014). When bone is decomposed or resorbed, it releases calcium, phosphorus, and other minerals in the blood circulation. Injecting LDN could enhance bone resorption, but not significantly decrease the number of osteogenic BMMSCs. Simultaneously, it downregulates the level of Smad1 protein.

Furthermore, we found that the medium level exercise attenuated the decreasing BMD, cortical bone volume, trabecular bone volume, Tb.N, and serum calcium levels caused by LDN injection. Different intensities of exercise training affected the serum calcium levels differentially. The medium level of exercise combined with LDN injection also increased the Smad1 protein expression as compared to LDN injection alone.

## Data Availability Statement

The original contributions presented in the study are included in the article/Supplementary Material inquiries can be directed to the corresponding authors.

## Ethics Statement

These mice in the study were purchased from the Second Military Medical University Animal Centre (Shanghai SLAC Laboratory Animal Co.) according to the protocols (Approval Number: 2007000563539) authorized by the Ethical Committee of Shanghai University of Sport on the Care and Use of Animal Subjects. We have complied with all relevant ethical regulations for animal testing and research.

## Author Contributions

JZ designed this study. LZ, WW, ZS, LL, and JF did the experiments. LZ and YY analyzed the experimental data. LZ and BG were responsible for manuscript writing. JZ revised the manuscript and supervised this entire program. All authors approved the final version of this manuscript.

## Conflict of Interest

The authors declare that the research was conducted in the absence of any commercial or financial relationships that could be construed as a potential conflict of interest.

## Supplementary Material

Supplementary Figure 1 Biomechanical parameters of bone. **(A)** Maximum load. **(B)** Yield stress. **(C)** elastic modulus.

## References

[B1] Anna L. Xuechun X. James Y. Huiyi K. Huijuan L. Yuji M. (2014). Pdgf-aa promotes osteogenic differentiation and migration of mesenchymal stem cell by down-regulating pdgfrα and derepressing bmp-smad1/5/8 signaling. *PLoS One* 9:e113785. 10.1371/journal.pone.0113785 25470749 PMC4254917

[B2] Carvalho D. C. Garlipp C. R. Bottini P. V. Afaz S. H. Moda M. A. Cliquet Jr A. (2006). Effect of treadmill gait on bone markers and bone mineral density of quadriplegic subjects. *Braz. J. Med. Biol. Res.* 39 1357–1363. 10.1590/s0100-879x2006001000012 17053843

[B3] Casazza K. Hanks L. J. Hidalgo B. Hu H. H. Affuso O. (2012). Short-term physical activity intervention decreases femoral bone marrow adipose tissue in young children: a pilot study. *Bone* 50 0–27. 10.1016/j.bone.2011.08.032 PMC3246551 21939791

[B4] Chen G. Deng C. Li Y. P. (2012). Tgf-β and bmp signaling in osteoblast differentiation and bone formation. *Int. J. Biol. Sci.* 8 272–288. 10.7150/ijbs.2929 22298955 PMC3269610

[B5] Di C. Ming Z. Mundy G. R. (2003). Bone morphogenetic proteins. *Growth Fact.* 22 233–241. 10.1080/08977190412331279890 15621726

[B6] Droste S. K. Gesing A. Ulbricht S. Müller M. B. Linthorst A. C. Reul J. M. (2003). Effects of long-term voluntary exercise on the mouse hypothalamic-pituitary-adrenocortical axis. *Endocrinology* 144 3012–3023. 10.1210/en.2003-0097 12810557

[B7] Ferreira J. C. Rolim N. P. Bartholomeu J. B. Gobatto C. A. Kokubun E. Brum P. C. (2007). Maximal lactate steady state in running mice: effect of exercise training. *Clin. Exp. Pharmacol. Physiol.* 34 760–765. 10.1111/j.1440-1681.2007.04635.x 17600553

[B8] Halling L. C. Englund U. H. Narisawa S. Millán J. L. Magnusson P. (2013). Isozyme profile and tissue-origin of alkaline phosphatases in mouse serum. *Bone* 53 399–408. 10.1016/j.bone.2012.12.048 23313280 PMC3593980

[B9] Hasegawa Y. Schneider P. Reiners C. (2001). Age, sex, and grip strength determine architectural bone parameters assessed by peripheral quantitative computed tomography (pQCT) at the human radius. *J. Biomech.* 34 497–503. 10.1016/s0021-9290(00)00211-6 11266673

[B10] Heldin C. H. Miyazono K. Ten D. P. (1997). Tgf-beta signalling from cell membrane to nucleus through smad proteins. *Nature* 390 465–471. 10.1038/37284 9393997

[B11] Huang H. Dou L. Song J. Luo J. (2018). Cbfa2t2 is required for bmp-2-induced osteogenic differentiation of mesenchymal stem cells. *Biochem. Biophys. Res. Commun.* 496 1095–1101. 10.1016/j.bbrc.2018.01.144 29378183

[B12] Huang T. H. Lin S. C. Chang F. L. Hsieh S. S. Liu S. H. Yang R. S. (2003). Effects of different exercise modes on mineralization, structure, and biomechanical properties of growing bone. *J. Appl. Physiol.* 95 300–307. 10.1152/japplphysiol.01076.2002 12611764

[B13] Iura A. Mcnerny E. G. Zhang Y. Kamiya N. Tantillo M. Lynch M. (2015). Mechanical loading synergistically increases trabecular bone volume and improves mechanical properties in the mouse when bmp signaling is specifically ablated in osteoblasts. *PLoS One* 10:e0141345. 10.1371/journal.pone.0141345 26489086 PMC4619208

[B14] Jabs E. W. Müller Ulrich Li Xiang (1993). A mutation in the homeodomain of the human msx2 gene in a family affected with autosomal dominant craniosynostosis. *Cell* 75 443–450. 10.1016/0092-8674(93)90379-5 8106171

[B15] James A. W. (2013). Review of signaling pathways governing msc osteogenic and adipogenic differentiation. *Scientifica* 2013 1–17. 10.1155/2013/684736 24416618 PMC3874981

[B16] Jovanovic V. Salti A. Tilleman H. Zega K. Jukic M. Zou H. (2018). Bmp/smad pathway promotes neurogenesis of midbrain dopaminergic neuronsin vivoand in human induced pluripotent and neural stem cells. *J. Neuroence* 38 1540–1517. 10.1523/JNEUROSCI.1540-17.2018 PMC5815451 29321139

[B17] Katagiri T. Tsukamoto S. (2013). The unique activity of bone morphogenetic proteins in bone: a critical role of the smad signaling pathway. *Biol. Chem.* 394 703–714. 10.1515/hsz-2012-0310 23324379

[B18] Kishimoto K. Lynch R. P. Reiger J. Yingling V. R. (2012). Short-term jump activity on bone metabolism in female college-aged nonathletes. *J. Sports Sci. Med.* 11 31–38. 24149120 PMC3737855

[B19] Komori T. (2019). Regulation of Proliferation, Differentiation and Functions of Osteoblasts by Runx2. *Int. J. Mol. Sci.* 20:1694. 10.3390/ijms20071694 30987410 PMC6480215

[B20] Kramer H. F. Witczak C. A. Fujii N. Jessen N. Taylor E. B. Arnolds D. E. (2006). Distinct signals regulate as160 phosphorylation in response to insulin, aicar, and contraction in mouse skeletal muscle. *Diabetes* 55 2067–2076. 10.2337/db06-0150 16804077

[B21] Lee M. H. Kim Y. J. Kim H. J. Park H. D. Kang A. R. Kyung H. M. (2003). Bmp-2-induced runx2 expression is mediated by dlx5, and tgf-beta 1 opposes the bmp-2-induced osteoblast differentiation by suppression of dlx5 expression. *J. Biol. Chem.* 278 34387–34394. 10.1074/jbc.m211386200 12815054

[B22] Lee Y. C. Cheng C. J. Bilen M. A. Lu J. F. Satcher R. L. Yu-Lee L. Y. (2011). Bmp4 promotes prostate tumor growth in bone through osteogenesis. *Cancer Res.* 71 5194–5203. 10.1158/0008-5472.can-10-4374 21670081 PMC3148283

[B23] Li M. Wu W. Tan L. Mu D. Zhu D. Wang J. (2015). Low-magnitude mechanical vibration regulates expression of osteogenic proteins in ovariectomized rats. *Biochem. Biophys. Res. Commun.* 465 344–348. 10.1016/j.bbrc.2015.07.154 26239658

[B24] Lin C. F. Huang T. H. Tu K. C. Lin L. L. Tu Y. H. Yang R. S. (2012). Acute effects of plyometric jumping and intermittent running on serum bone markers in young males. *Eur. J. Appl. Physiol.* 112 1475–1484. 10.1007/s00421-011-2108-8 21837450

[B25] Lowery J. W. Rosen V. (2012). Allele-specific rna interference in fop silencing the fop gene. *Gene Therapy* 19 701–702. 10.1038/gt.2011.190 22130446

[B26] Magni P. Macchi C. Sirtori C. R. Corsi Romanelli M. M. (2016). Osteocalcin as a potential risk biomarker for cardiovascular and metabolic diseases. *Clin. Chem. Lab. Med.* 54 1579–1587. 26863345 10.1515/cclm-2015-0953

[B27] Mehl K. A. Davis J. M. Clements J. M. Berger F. G. Pena M. M. Carson J. A. (2005). Decreased intestinal polyp multiplicity is related to exercise mode and gender in apcmin/+ mice. *J. Appl. Physiol.* 98 2219–2225. 10.1152/japplphysiol.00975.2004 15894538

[B28] Monika M. Smieszek A. Klaudia C. Katarzyna B. Krzysztof M. (2015). Physical activity increases the total number of bone-marrow-derived mesenchymal stem cells, enhances their osteogenic potential, and inhibits their adipogenic properties. *Stem Cells Int.* 2015 1–11. 10.1155/2015/379093 26167185 PMC4488015

[B29] O’Kane J. W. Hutchinson E. Atley L. M. Eyre D. R. (2006). Sport-related differences in biomarkers of bone resorption and cartilage degradation in endurance athletes. *Osteoarthr. Cartil.* 14 71–76. 10.1016/j.joca.2005.08.003 16188465

[B30] Orcy R. Antunes M. F. Schiller T. Seus T. Böhlke M. (2014). Aerobic exercise increases phosphate removal during hemodialysis: a controlled trial. *Hemodial. Int.* 18 450–458. 10.1111/hdi.12123 24438516

[B31] Pacheco-Costa R. Davis H. M. Atkinson E. G. Dilley J. E. Byiringiro I. Aref M. W. (2018). Reversal of loss of bone mass in old mice treated with mefloquine. *Bone* 114 22–31. 10.1016/j.bone.2018.06.002 29879544 PMC6056320

[B32] Petzinger G. M. Walsh J. P. Akopian G. Hogg E. Abernathy A. Arevalo P. (2013). Effects of treadmill exercise on dopaminergic transmission in the 1-methyl-4-phenyl-1,2,3,6-tetrahydropyridine-lesioned mouse model of basal ganglia injury. *J. Neurosci.* 27 5291–5300. 10.1523/jneurosci.1069-07.2007 17507552 PMC6672356

[B33] Potier E. Noailly J. Ito K. (2010). Directing bone marrow-derived stromal cell function with mechanics. *J. Biomech.* 43 807–817. 10.1016/j.jbiomech.2009.11.019 19962149

[B34] Riddle R. C. Taylor A. F. Genetos D. C. Donahue H. J. (2005). Map kinase and calcium signaling mediate fluid flow-induced human mesenchymal stem cell proliferation. *AJP Cell Physiol.* 290 C776–C784. 10.1152/ajpcell.00082.2005 16267109

[B35] Rodan G. A. (1998). Bone homeostasis. *Proc. Natl. Acad. Sci. U S A.* 95 13361–13362. 9811806 10.1073/pnas.95.23.13361 PMC33917

[B36] Ronga M. Fagetti A. Canton G. Paiusco E. Surace M. F. Cherubino P. (2013). Clinical applications of growth factors in bone injuries: experience with bmps. *Inj. Int. J. Care Inj.* 44 S34–S39. 10.1016/S0020-1383(13)70008-1 23351868

[B37] Roussignol X. Currey C. Duparc F. Dujardin F. (2012). Indications and results for the exogen^TM^ ultrasound system in the management of non-union: a 59-case pilot study. *Orthopaed. Traumatol. Surg. Res.* 98 206–213. 10.1016/j.otsr.2011.10.011 22424956

[B38] Shen C. L. Williams J. S. Chyu M. C. Paige R. L. Stephens A. L. Chauncey K. B. (2007). Comparison of the effects of tai chi and resistance training on bone metabolism in the elderly: a feasibility study. *Am. J. Chin. Med.* 35 369–381. 10.1142/s0192415x07004898 17597496

[B39] Shin J. W. Swift J. Ivanovska I. Spinler K. R. Buxboim A. Discher D. E. (2013). Mechanobiology of bone marrow stem cells: from myosin-ii forces to compliance of matrix and nucleus in cell forms and fates. *Differentiation* 86 77–86. 10.1016/j.diff.2013.05.001 23790394 PMC3964600

[B40] Smith E. L. Gilligan C. (1991). Physical activity effects on bone metabolism. *Calcified Tissue Int.* 49 S50–S54. 10.1007/BF02555089 1933599

[B41] Smith S. M. Abrams S. A. Davisstreet J. E. Heer M. O’Brien K. O. Wastney M. E. (2014). Fifty years of human space travel: implications for bone and calcium research. *Annu. Rev. Nutr.* 34 377–400. 10.1146/annurev-nutr-071813-105440 24995691

[B42] Smith S. M. Zwart S. R. Heer M. Lee S. M. C. Baecker N. Meuche S. (2008). Wise-2005: supine treadmill exercise within lower body negative pressure and flywheel resistive exercise as a countermeasure to bed rest-induced bone loss in women during 60-day simulated microgravity. *Bone* 42 572–581. 10.1016/j.bone.2007.11.015 18249055

[B43] Sumida S. Iwamoto J. Uenishi K. Otani T. (2014). One-year changes in bone mineral density and bone turnover markers in premenopausal amateur runners: a prospective study. *Keio J. Med.* 63 43–51. 10.2302/kjm.2013-0010-oa 24920066

[B44] Tadic T. Dodig M. Erceg I. Marijanovic I. Mina M. Kalajzic Z. (2002). Overexpression of dlx5 in chicken calvarial cells accelerates osteoblastic differentiation. *J. Bone Miner. Res.* 17 1008–1014. 10.1359/jbmr.2002.17.6.1008 12054155

[B45] Tong M. L. Lee E. H. (2013). Transcriptional regulatory cascades in runx2-dependent bone development. *Tissue Engine. Part B Rev.* 19 254–263. 10.1089/ten.teb.2012.0527 23150948 PMC3627420

[B46] Tucker L. A. Strong J. E. Lecheminant J. D. Bailey B. W. (2015). Effect of two jumping programs on hip bone mineral density in premenopausal women: a randomized controlled trial. *Am. J. Health Promot.* 29 158–164. 10.4278/ajhp.130430-quan-200 24460005

[B47] Vesper H. W. Demers L. M. Eastell R. Garnero P. Kleerekoper M. Robins S. P. (2002). Assessment and recommendations on factors contributing to preanalytical variability of urinary pyridinoline and deoxypyridinoline. *Clin. Chem.* 48 220–235. 10.1093/clinchem/48.2.220 11805003

[B48] Wang L. You X. Lotinun S. Zhang L. Wu N. Zou W. (2020). Mechanical sensing protein PIEZO1 regulates bone homeostasis via osteoblast-osteoclast crosstalk. *Nat. Commun.* 11:282. 10.1038/s41467-019-14146-6 PMC6962448 31941964

[B49] Wang Y. Newman M. R. Benoit D. S. W. (2018). Development of controlled drug delivery systems for bone fracture-targeted therapeutic delivery: a review. *Eur. J. Pharmaceut. Biopharmaceut.* 127 223–236. 10.1016/j.ejpb.2018.02.023 29471078 PMC5948156

[B50] Winther A. Dennison E. Ahmed L. A. Furberg A. S. Grimnes G. Jorde R. (2014). The tromsø study: fit futures: a study of norwegian adolescents’ lifestyle and bone health. *Arch. Osteopor.* 9:185. 10.1007/s11657-014-0185-0 24893722

[B51] Woitge H. W. Friedmann B. Suttner S. Farahmand I. Müller M. Schmidt-Gayk H. (1998). Changes in bone turnover induced by aerobic and anaerobic exercise in young males. *J. Bone Miner. Res.* 13 1797–1804. 10.1359/jbmr.1998.13.12.1797 9844096

[B52] Yu P. B. Deng D. Y. Lai C. S. Hong C. C. Cuny G. D. Bouxsein M. L. (2008a). Bmp type i receptor inhibition reduces heterotopic [corrected] ossification. *Nat. Med.* 14 1363–1369. 10.1038/nm.1888 19029982 PMC2846458

[B53] Yu P. B. Hong C. C. Sachidanandan C. Babitt J. L. Deng D. Y. Hoyng S. A. (2008b). Dorsomorphin inhibits bmp signals required for embryogenesis and iron metabolism. *Nat. Chem. Biol.* 4 33–41. 10.1038/nchembio.2007.54 18026094 PMC2727650

[B54] Yu Y. Lin Y. Yang G. Tian L. (2017). The interplay between tgf-β/smad and bmp/smad signaling pathways in the epithelial mesenchymal transition of a549 cells induced by silica. *Toxicol. Mech. Methods* 28 286–292. 10.1080/15376516.2017.1407978 29161937

[B55] Zhang J. Cai L. Tang L. Zhang X. Yang L. Zheng K. (2018). Highly dispersed lithium doped mesoporous silica nanospheres regulating adhesion, proliferation, morphology, alp activity and osteogenesis related gene expressions of bmscs. *Colloids Surf. B Biointerfaces* 170 563–571. 10.1016/j.colsurfb.2018.06.038 29975904

[B56] Zhang L. Chen X. Wu J. Yuan Y. Guo J. Biswas S. (2017). The effects of different intensities of exercise and active vitamin D on mouse bone mass and bone strength. *J. Bone Miner. Metab.* 35 265–277. 10.1007/s00774-016-0764-9 27357401

[B57] Zhang L. Liu W. Zhao J. Ma X. Shen L. Zhang Y. (2016). Mechanical stress regulates osteogenic differentiation and rankl/opg ratio in periodontal ligament stem cells by the wnt/β-catenin pathway. *Biochimica Biophysica Acta* 1860:2211–2219. 10.1016/j.bbagen.2016.05.003 27154288

