# Supplementary figures and images for: Medium-Intensity Treadmill Exercise Exerts Beneficial Effects on Bone Modeling Through Bone Marrow Mesenchymal Stromal Cells
Source: Front Cell Dev Biol. 2020 Nov 24;8:600639. doi: 10.3389/fcell.2020.600639 (PMC7732523; doi:10.3389/fcell.2020.600639)

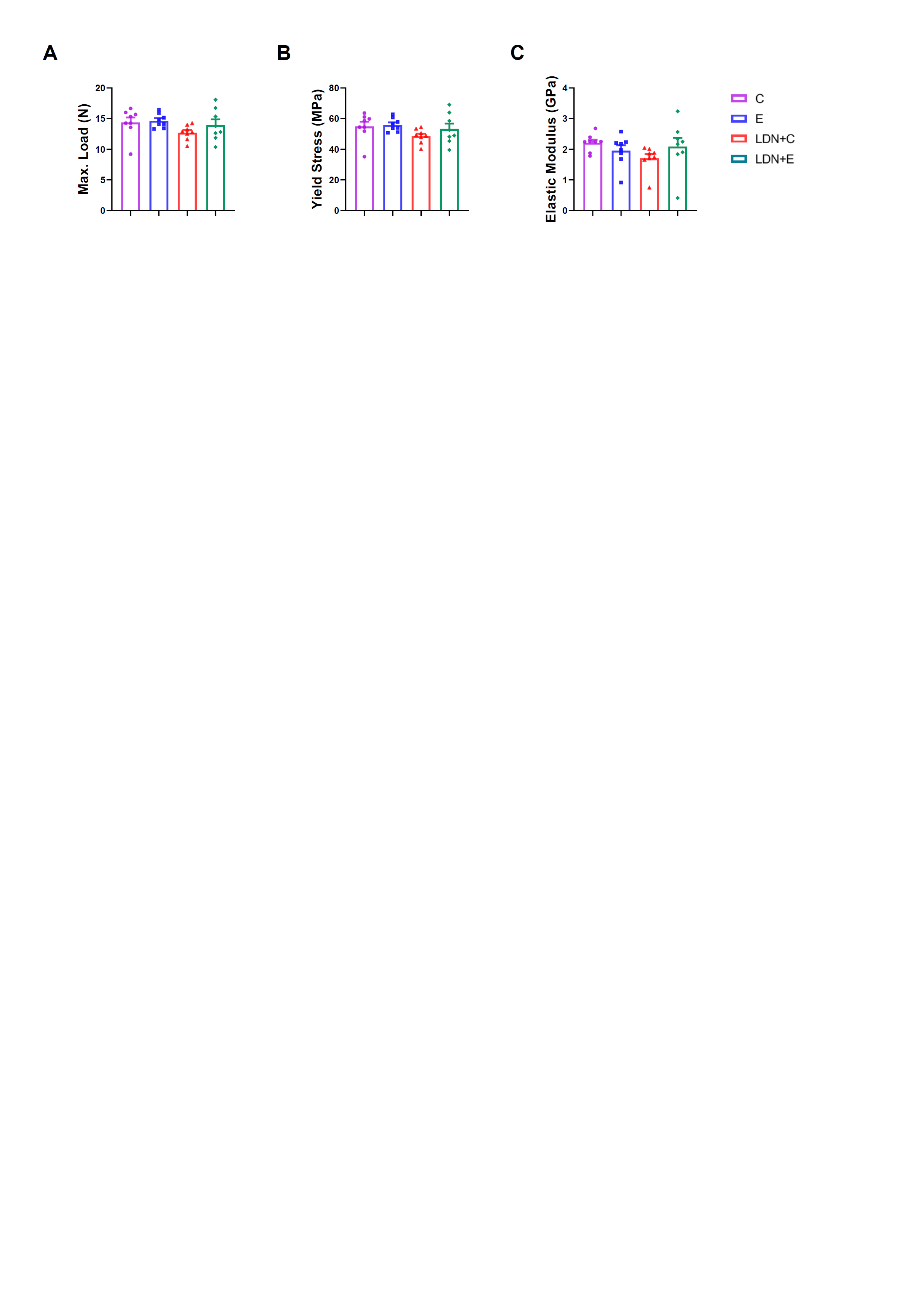

Supplement: Supplementary Figure 1 — Biomechanical parameters of bone. (A) Maximum load. (B) Yield stress. (C) elastic modulus. [file Image_1.TIF]
